# Treatment Outcomes of Patients with Squamous Cell Carcinoma of the Vulva: The Largest Series from a Tertiary Care Hospital

**DOI:** 10.1155/2018/4723167

**Published:** 2018-09-03

**Authors:** Panida Meelapkij, Prapaporn Suprasert, Orthai Baisai

**Affiliations:** Division of Gynecologic Oncology, Department of Obstetrics and Gynecology, Chiang Mai University, Chiang Mai, Thailand

## Abstract

**Objective:**

To evaluate the outcomes of squamous cell carcinoma (SCCA) of the vulva treated at our tertiary care center.

**Methods:**

The medical records of SCCA patients treated between January 2006 and December 2015 were retrospectively reviewed.

**Results:**

One hundred forty-five patients met the criteria with the median age of 57 years old, and 58.6% had an underlying disease. The distribution of stages was as follows: IA 6.2%, IB 21.4%, II 26.2%, IIIA 14.5%, IIIB 6.2%, IIIC 9.7%, IVA 9.0%, and IVB 6.9%. One hundred and nine patients underwent surgical intervention and radical local excision with bilateral groin node dissection as the most frequent procedure. Approximately half of the patients received combined treatment with surgery followed by radiation with or without chemotherapy. Recurrence developed in 127 patients after the median follow-up time of one year with the common sites in the groin and vulva region. However, no significant difference in survival occurred in patients with and without groin node recurrence (15 vs. 28 months, *P*=0.109). The five-year overall survival was 50.8%.

**Conclusions:**

The survival of patients with SCCA vulvar cancer was modest. The common failure sites were groin and vulva regions with unfavorable outcomes.

## 1. Introduction

Vulva carcinoma is a rare tumor demonstrated in only four percent of all gynecologic malignancies. The overall incidence is between two and seven cases per 100,000 women, and over 90% of this rare disease are related to squamous cell carcinoma (SCCA) histology that could be classified into two types [1]. The first is associated with human papilloma virus (HPV) infection that causes 86.7% of vulvar intraepithelial neoplasia (VIN) and 28.6% in invasive vulvar cancer from the study of the worldwide HPV genotype attribution in over 2000 cases of VIN and vulvar cancer [2]. The second one is HPV-independent type that causes vulvar nonneoplastic epithelial disorders such as vulvar dystrophy or lichen sclerosis. This second type often occurs in elderly patients [3, 4]. Frequent symptoms of vulvar cancer are chronic pruritus, vulvar bleeding, dysuria, abnormal discharge, and pain with the most obvious clinical manifestation of a mass with or without ulcer [3]. The main treatment especially in an early stage is radical or wide local excision and bilateral inguinofemoral lymphadenectomy in patients whose depth of tumor invasion measures more than one mm. The aim of the vulva excision is to remove the entire lesion with tumor-free margin of at least one cm. Radiation of the pelvis that includes the groin region and vulvar area is added when the tumor involves a groin node or vulvar margin, respectively [3]. For patients with advanced stages, the treatment is individualized depending on the extent of the primary lesion and the performance status of the patients. Some physicians preferred primary radiation to the groin, pelvis, and vulva with or without chemotherapy or treated with neoadjuvant chemotherapy followed by surgery [3].

In Chiang Mai University Hospital, a Northern Tertiary Care Hospital, SCCA of the vulva was found in an average of ten cases a year. With the rarity of this disease, the outcomes of treatment especially in a single institute are still limited. We conducted this retrospective study to evaluate the survival outcomes of SCCA vulva cancer treated at our center. These results should be beneficial for improving the knowledge and treatment of this rare disease.

## 2. Materials and Methods

After the protocol was approved by the local ethics committee, the medical records of the patients with SCCA vulva cancer treated at Chiang Mai University Hospital between January 2006 and December 2015 were retrospectively reviewed. The patients who had been previously treated with other cancers were excluded. All pathological specimens were reviewed by our gynecologic pathologists. Generally, it has been our practice to perform surgery with radical local excision (RLE) or wide local excision (WLE) and bilateral groin node dissection (BGND) that included inguinofemoral node removal in operable patients. However, some of the patients with unresectable vulvar lesion only underwent a BGND. The adjuvant radiation or concurrent chemoradiation (CCRT) of the pelvis and groin regions was administered to patients with positive groin nodes. Vulva radiation was also administered in patients who had positive margins or presented with tumor-free margin less than 8 mm. In patients with a locally advanced stage, whole pelvic plus groin radiation or CCRT with or without BGND were the primary treatments while platinum-based chemotherapy was the main treatment in patients with distant metastasis or recurrent setting. Neoadjuvant chemotherapy was given in patients with a large tumor size before surgery or given in patients whose schedule for starting radiation or CCRT was longer than three to four weeks.

After completion of treatment, the patients were routinely followed up with gynecologic oncologists for physical and pelvic examinations every three months in the first year, every four months in the second year, and every six months in the third to fifth years, then annually. The patients were diagnosed as recurrent by physical examination combined with the appropriate imaging.

The following information was included: (1) clinical data, (2) Figo 2009 staging, (3) primary treatment methods, (4) sites of recurrence, (5) progression-free survival (PFS) defined as the time from the initial treatment to the time of recurrence or last contact, and (6) overall survival (OS) defined as the same start time as PFS to the time of patient death or the time that the patient was still alive at the time of the collection from the last data search from the Thai Civil Registration.

Statistical analysis of the data was carried out using IBM SPSS statistics for Window program (Version 22). Descriptive data of all studied patients were presented as means or median with range and discrete data reported as number and percentages. The PFS and OS were estimated by the Kaplan–Meier method. Factors influencing survival were analyzed using log-rank test analysis. Statistical significance was noted when a *P* value was less than 0.05.

## 3. Results

In the study period, there were 172 patients with vulvar cancer. Of these patients, 27 cases were excluded due to non-SCCA histology (23 cases) and those previously diagnosed as cervical cancer (four cases). Finally, 145 cases met the inclusion criteria and were recruited into this study. The basic clinical data were noted in Table 1. The median age of the studied patients was 57 years of age, and nearly 70% were menopausal. Fourteen patients (9.6%) presented at less than or equal to 40 years old. Three-fourths of the patients had no previous history of operation, and 60% presented with underlying disease. Nearly half of them were in Stages I and II, and the median tumor size surface area was 12 cm^2^. The maximum size of right and left groin nodes was 6 cm and 12 cm, respectively.

One hundred and nine patients (73%) received surgical intervention. The type of operation in each stage is shown in Table 2. Almost half of the operations were RLE with BGND and with the majority of the operations in Stages IB to IIIA. The second most frequent operation at 10% was BGND with and without wide local excision (WLE). In four cases with Stage IVB who received surgery, three cases underwent pelvic node operation which were found to be metastasized and the remaining one had a positive pelvic node from the computer tomography (CT) scan. This case received neoadjuvant chemotherapy with two cycles of cisplatin plus 5-fluorouracil followed by concurrent chemoradiation of the pelvis before undergoing RLE with BGND to remove the residual primary tumor and for groin node evaluations. One case in Stage IA received vulvar reconstruction after an anterior vulvectomy due to her large tumor size of 5 × 5 cm. This case had SCCA arising in Paget's disease, and the width and depth of SCCA area was 1.7 mm and 0.6 mm, respectively. In addition, perioperative morbidities occurred in 56 cases (51.4%) that consisted of fever (19), urinary tract infection (2), infected wound (28), lymphocyst (5), lymphedema (5), and left hemiparalysis with myocardial ischemia (1).

Regarding primary treatment, the details are presented in Table 3. About half of the studied patients received combination treatment with surgery followed by radiation, whereas the single treatment with surgery, radiation, and CCRT was performed in 17.2%, 9.0%, and 10.3%, respectively. Only two patients were operated after chemotherapy and CCRT in each. Neoadjuvant chemotherapy (NAC) was administered in 27 cases (18.6%) with the following various regimens: cisplatin plus 5-fluorouracil (20 cases), mitomycin C plus 5-fluorouracil (three cases), cisplatin (three cases), and carboplatin (one case) while the subsequent treatments underwent surgery (four cases), surgery and radiation (nine cases), surgery and CCRT (two cases), CCRT (11 cases), and CCRT and surgery (one case).

Table 4 demonstrates the outcomes of treatment. Only 127 patients were evaluated while the rest could not be assessed due to loss to follow-up in 14 cases and initial palliative treatment in four cases. The recurrence rate was found in 45 cases (35.4%) with the highest rate in Stage IVB. The median recurrence-free survival was 12 months (1–99 months). The most common recurrence sites were in the groin node and vulvar regions. In patients with initial Stage IB and II with no previous groin involvement, recurrence occurred in groin nodes in three from 30 cases (10.0%) and four from 33 cases (12.1%), respectively. Moreover, in patients with an initial presentation of groin node involvement (Stage III), even though they received whole pelvic radiation, groin recurrence still occurred in 28.2%. Further treatment after recurrence consisted of palliation (13), surgery (4), chemotherapy (13), CCRT (5), and debulking of the tumor with vulva reconstruction followed by radiation (1).

**Figure 1:**
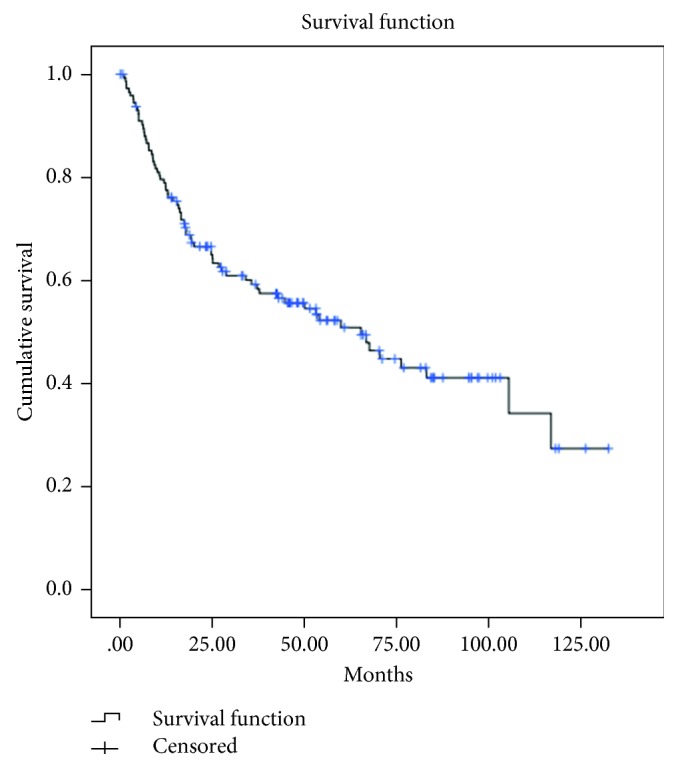
Estimated overall survival (*N* = 145). Median overall survival = 59 months (1–132 months), 5-year overall survival = 50.8%, death = 72 (49.7%), and alive = 73 (50.3%).

**Figure 2:**
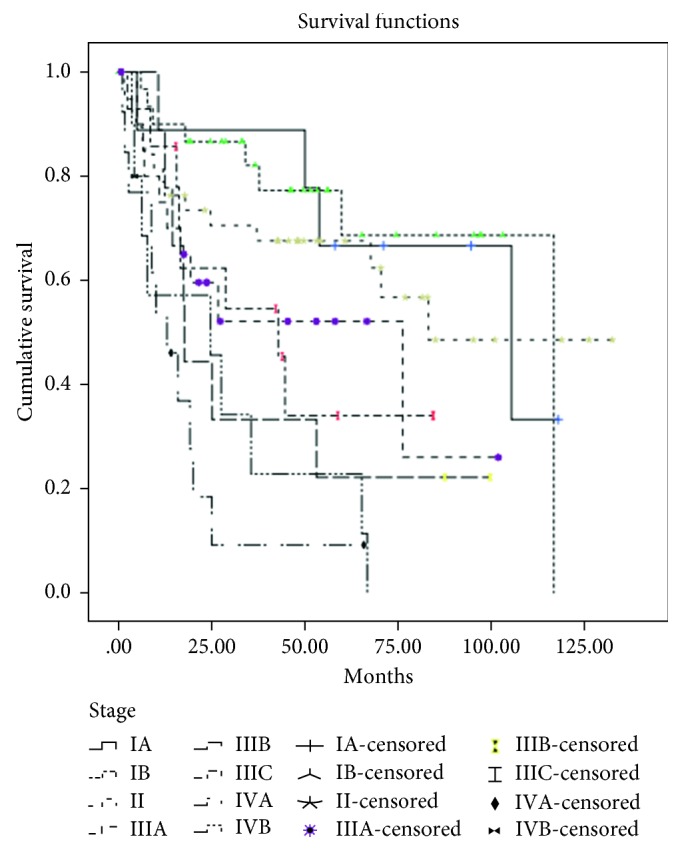
Estimated overall survival divided by stage.

Concerning overall survival, the Kaplan survival curve is demonstrated in Figure 1. About half of the studied patients died, and the median overall survival was 59 months with a five-year overall survival rate of 50.8%. The estimated five-year overall survival rate in each stage was as follows: Stage IA was 66.7%, IB was 68.7%, II was 67.6%, IIIA was 52.1%, IIIB was 22.2%, IIIC was 34.1%, IVA was 9.2%, and IVB was 11.4%. These differences were statistically significant with a *P* value < 0.001, as shown in Figure 2. Furthermore, in 45 cases that developed recurrence, the median overall survival in patients with or without groin recurrence was five months and 23 months after recurrence, respectively (*P*=0.049).

Among nine patients in stage IA, four of them died from other causes and only one case developed local recurrence one month after undergoing wide local excision and received further vulvar surgery with complete resection. This case also died with other causes four years after treatment.

## 4. Discussion

SCCA is the most common histology of vulvar cancer. In our institute, SCCA presented as high as 86.6% of all vulvar cancers. The median age of our patients was 57 years which was lower than the German data that revealed the mean age of their patients of 72 years [5]. However, many recent publications showed the tendency of increased incidence rates of vulvar cancer in younger women [6–9]. Therefore, the median age of our patients and in the current reports was in a range of 55–60 years [10, 11]. One possible explanation regarding the tendency of developing vulvar cancer in younger women in this era is the increased high-risk human papillomavirus (HPV) infections that cause VIN, the known major predisposing factor for vulva cancer [1–3]. To support this concept, Siriaunkgul et al. [12] previously published a study about HPV in 47 northern Thai women with vulvar cancer by using a polymerase chain reaction method to detect HPV DNA from paraffin-embedded samples. They revealed that HPV infection was detected at 62% and Type 16 was the highest frequency type, and no significant difference was shown in tumor stage distribution regarding the status of HPV infection. The high prevalence of HPV infection among Thai vulvar cancer patients was unclear. It may be possibly due to the limited number of patients, the nature of Asian population and the detection technique. In the present study, we found 9.6% of the studied patients were less than or equal to 40 years of age which was less than a previous study from the United States that showed the prevalence in this age group as high as 15% [13]. However, that prevalence was from many institutes in the United States while our data came from a single institute.

About the stage distribution, nearly half of the studied patients presented in a locally advanced stage which was like the nationwide population-based study from Denmark that found over 60% of vulva cancer patients to be in a locally advanced stage. The possible reason might be due to neglect from both patients and the physicians [7].

In the present study, the main primary lesion surgical procedure was a conservative trend that found safe removal through an RLE with the aim to get a one to two cm tumor-free margin about 1-2 cm excised deeply at the level of the perineal membrane instead of a total vulvectomy [14, 15]. In addition, some patients underwent WLE that removed the primary lesion with tumor-free margin similar to RLE but with a different depth level. WLE excised at the level of the subcutaneous layer. This procedure has been accepted in many centers [16].

Regarding groin node dissection, BGND that removed both superficial and deep groin nodes was the most frequent procedure in our patients. Removal of only superficial groin nodes is not appropriate due to the high recurrence of groin nodes when compared to removal of groin nodes in both layers [17]. However, BGND was accepted for omission in Stage IA because groin node involvement in this stage is very rare [15]. Meanwhile, unilateral groin node dissection could be safely done in patients with tumors in the lateral area with uninvolved ipsilateral groin nodes. This concept came from previous knowledge that found no contralateral groin node metastasis in such cases [15]. Three important issues remain unclear. Firstly, the definition of laterality that did not define the distance from the midline of the vulva area is unclear. Secondly, the tumor size was unclear to be less than two or three cm. Thirdly, for invasion cutoff, a depth of <3 mm has been proposed but it still showed contralateral risk of nodal metastasis at less than 1% [15, 18]. Thus, unilateral groin node dissection in our series was only performed in one case. However, about two-thirds of patients that underwent inguinofemoral lymphadenectomy were associated with high rates of postoperative complications especially lymphedema and lymphocyst. Therefore, sentinel lymph node biopsy was recently proposed in early stage patients with clinically negative groin nodes to reduce these complications [19]. Previous studies showed that this procedure could be reliable and safe in the early stages, but still required special equipment and the expertise of surgeons and pathologists [20, 21]. In our institute, sentinel node biopsy was not routinely done.

Adjuvant treatment with radiation after radical vulvectomy and BGND has proven to significantly reduce local relapse and decreased cancer-related deaths from GOG 37 study in patients with positive groin nodes [22]. In addition, a Surveillance, Epidemiology, and End Result (SEER) analysis indicated that for single groin node metastasis, adjuvant radiation improved disease-specific survival and increased overall survival if less than 12 lymph nodes were removed [23]. Furthermore, the benefit of adjuvant radiation also was proved in patients with one intracapsular macrometastasis node (>2 mm) [24]. However, even giving adjuvant radiotherapy in those patients with node-positive, the data from the AGO-CaRE-1 Study, a large multicenter study from 29 gynecologic cancer centers in Germany, still found poorer survival outcomes in node-positive patients who received adjuvant radiotherapy when compared to node-negative patients [25]. The authors suggested the role of chemoradiation in these node-positive patients. In the same year, Gill et al. published the National Cancer Database of the United States with the aim to analyze the benefit of adding chemotherapy to adjuvant radiation in node-positive vulvar cancer. They found adjuvant chemotherapy resulted in a 38% reduction in the risk of death [26]. In the present study, adjuvant radiation to the pelvis including the groin region was given in patients with groin node-positive, even with only one node and chemotherapy was administered in the setting of CCRT in some cases. However, a high groin recurrence was found in these patients. This might be from the various reasons such as a long duration time between surgery and start of radiotherapy usually more than one month in our patients and the biology of our tumor cells. Thus, new treatment strategies should be investigated.

For adjuvant radiation treatment in patients with close or positive surgical margins, Ignatov et al. [27] recently revealed that the five-year overall survival of these patients increased to 67.6% in cases that received radiation directly to the vulvar region compared to only 29% of cases who did not receive such treatment. In the present study, 64 patients were in Stages I-II and 40 of these (62.5%) received adjuvant radiation to their vulvar site due to positive and close margins (<8 mm). The high percentage of adjuvant radiotherapy in these patients might be from a large initial tumor size in our patients. Thus, most of them revealed the tumor-free margin less than 8 mm and received vulva radiation.

In locally advanced stages, neoadjuvant chemotherapy (NAC) has been mentioned in this era to reduce the primary size and decrease micrometastasis [19]. In our series, NAC was given in 18.6% of the patients. However, the principle treatment of these stages was radiation with or without chemotherapy. In some cases, BGND was performed to remove the positive groin nodes before giving radiation which had been proven from GOG 37 to show benefits as mentioned above [23].

Concerning the outcome of treatment, the recurrence rate occurred in 35% of the follow-up patients with the median recurrence rate at one year with the groin nodes being the most frequent recurrence site. This recurrence rate corresponded to the previous reports that were in a range of 12–37%, most of them occurring within two years [28]. The independent risk factors for local recurrences are a higher age, a multifocal tumor, a depth of invasion more than two mm, lymphovascular space invasion, and the presence of groin node dissection [29]. Unfortunately, the present paper did not have available data in the recurrence patients except initial groin metastasis that was classified as Stage III.

Furthermore, in patients without groin node involvement, our study found that seven of 63 patients (11.1%) experienced groin node recurrence. This recurrence rate was higher than the previous reports that found a groin recurrence rate of 2.9–4.9% [30, 31]. An explanation of this high groin recurrence rate in our study may be from two reasons. Firstly, the surgical technique might not be more extensive to remove all inguinofemoral nodes. Gadducci et al. [31] found that groin recurrence in node-negative patients as high as 12% when the number of removed nodes was less than 12. Unfortunately, the data of the number of removed nodes in our study were not available. However, a high groin node recurrence at 17% also occurred in an Egyptian study [24] which performed only superficial groin node dissection in Stages I-II. Thus, the more extensive removal of superficial and deep groups of groin nodes should improve our outcomes. Secondly, there is undetected nodal micrometastasis of our patients. In the present study, the conventional histologic evaluation method was used to detect lymph node status. This might have missed the micrometastasis. Derdelis et al. [32] reported that about 5% of initial node-negative patients were found with micrometastasis when using an ultrastaging technique.

Regarding the survival outcome, the five-year overall survival in the present study was 50.8% that was in the lower range of the literature publications which revealed a 5-year overall survival in a range of 50–90% [29]. In addition, the survival in each stage of the present study was lower than the recent reports [33, 34]. Even in stage IA, our results revealed the estimated five-year overall survival rate at only 66.7%. This rate was lower than a previous publication that reported an excellent outcome at 92% [33]. The unsatisfactory outcome of our study might be from the lower number of observed cases and the inclusion of noncancer-specific deaths. Moreover, in recurrence patients, groin recurrence showed a nearly significantly worse survival outcome than another recurrence site. The median overall survival at five months of our patients with groin recurrence was shorter than the recent Italian study which revealed a median overall survival at nine months after recurrence [35]. This poorer outcome might be explained from the lower percentage of our patients who received further treatment after developing recurrence when compared to Italian study (50% vs. 80%).

In addition, another explanation for the poor outcomes of the present study is possibly from the limitations of the data for cancer-specific survival. Twenty percent of the studied patients were lost to follow-up, and most of these patients were elderly and had underlying disease. Thus, death from other causes was probable. Besides this, the differences in ethnicity might be influenced by the immunohistochemistry such as P16 and P53 mutations that were previously found to be the independent prognostic factors [36]. Unfortunately, we did not have any data regarding this issue. Therefore, further investigation of these aspects should be pursued. However, with the optimal number of studied patients, the results of the present study reflected the treatment outcomes from a single tertiary care hospital and will be beneficial to improve further management for this rare disease.

In conclusion, the survival of patients with vulvar cancer in Thailand was modest especially in the advanced cases. The most common failure site was the groin region, and when it occurred, a very poor prognosis with a short survival time was the main problem even though further treatment was received.

## Figures and Tables

**Table 1:** Basic clinical data (*N* = 145).

Data	*N* (%)
Median age (range)	57.0 (31–86)
Median body mass index (range)^*∗*^	21.78 (11.74–34.67)
Median tumor size surface area (range: cm^2^)	12.00 (0.10–216.0)
Presented with underlying disease^*∗∗∗*^	85 (58.6)
Stage	
IA	9 (6.2)
IB	31 (21.4)
II	38 (26.2)
IIIA	21 (14.5)
IIIB	9 (6.2)
IIIC	14 (9.7)
IVA	13 (9.0)
IVB	10 (6.9)
Median length of stay (range: days)	15.0 (4–104)

^*∗*^BMI missing 18 cases. ^*∗∗*^Measurement of the largest node from physical examination or imaging, right side missing 3 cases and left side missing 4 cases. ^*∗∗∗*^Underlying disease: diabetic mellitus (DM) (7), hypertension (HT)(15), dyslipidemia (DLP)(3), coagulopathy (1), anemia (1), DM and HT (4), DM and DLP (8), DM, HT, and DLP (8), HIV (13), old pulmonary TB (3), thyrotoxicosis (3), and others (18).

**Table 2:** Types of surgery divided by stage (*N* = 109).

Operation	Stage								Total (%)
	IA	IB	II	IIIA	IIIB	IIIC	IVA	IVB	
RLE with BGND	—	20	19	10	1	7	1	1	59 (54.1)
RLE with unilateral GND	—	4	1	—	—	1	—	—	6 (5.5)
RLE with BGND with BPNS	—	—	2	1	1	—	—	2	6 (5.5)
WLE	6	—	—	2	—	—	—	—	8 (7.3)
WLE with BGND	1	1	2	1	4	2	—	—	11 (10.2)
WLE with unilateral GND	—	—	—	—	—	1	—	—	1 (0.9)
Biopsy at groin node and left upper labia minora	—	—	—	—	—	—	1	—	1 (0.9)
BGND	—	2	3	1	2	1	2	—	11 (10.2)
BGND + BPND	—	1	1	—	—	—	—	1	3 (2.7)
Anterior vulvectomy with fasciocutaneous flap with rectus sheath	1	—	—	—	—	—	—	—	1 (0.9)
Left hemivulvectomy	—	—	—	1	—	—	—	—	1 (0.9)
TAH with BSO with anterior vulvectomy with BGND∗	—	—	—	—	—	1	—	—	1 (0.9)
Total	8	28	28	16	8	13	4	4	109 (100)

^*∗*^TAH with BSO due to ovarian dermoid cyst. RLE = radical local excision; BGND = bilateral groin node dissection; BPNS = bilateral pelvic node sampling; WLE = wide local excision; GND = groin node dissection; BPND = bilateral pelvic node dissection; TAH = transabdominal hysterectomy; BSO = bilateral salpingo-oophorectomy.

**Table 3:** Primary treatment divided by stage.

Treatment	Stage								Total (%)
	IA	IB	II	IIIA	IIIB	IIIC	IVA	IVB	
Supportive treatment	—	1	2	—	—	—	1	—	4 (2.7)
Surgery	6	11	7	1	—	—	—	—	25 (17.2)
Radiation	1	—	5	3	—	—	3	1	13 (9.0)
CCRT	—	2	3	2	1	—	4	3	15 (10.3)
Chemotherapy	—	—	—	—	—	1	1	2	4 (2.7)
Surgery followed by radiation	2	17	21	11	6	9	1	2	69 (47.6)
Surgery followed by CCRT	—	—	—	3	2	4	2	1	12 (8.3)
Surgery followed by chemotherapy	—	—	—	—	—	—	1	—	1 (0.7)
CCRT followed by surgery primary lesion	—	—	—	1	—	—	—	1	2 (1.5)
Total	9	31	38	21	9	14	13	10	145 (100)

CCRT = concurrent chemoradiation.

**Table 4:** Outcomes divided by stage (*N* = 127).

Stage	Outcome			Total (%)
	None	Recurrence (%)	Recurrent sites (N)	
IA	8	1 (11.1)	Vulva (1)	9 (7.0)
IB	25	5 (17.2)	Groin node (3), vulva (2)	30 (23.5)
II	23	10 (31.3)	Groin node (4), vulva (6)	33 (25.9)
IIIA	12	7 (36.8)	Groin node (3), vulva(1), vulva and groin node (1), groin node and skin (1), lung and supraclavicular node (1)	19 (14.9)
IIIB	5	2 (28.6)	Groin node (1), vulva (1)	7 (5.4)
IIIC	5	8 (61.5)	Groin node (4), vulva (3), vulva and groin node (1)	13 (10.2)
IVA	3	6 (66.7)	Groin node (4), vulva (2)	9 (7.0)
IVB	2	6 (75.0)	Lung (1), liver (1), breast (1), vulva (1), groin node and skin (2)	8 (6.1)
Total	82	45 (35.4)	Vulva (17), groin node (19), vulva and groin node (2), groin node and skin (3), lung and supraclavicular node (1), lung (1), liver (1), breast (1)	127 (100)

Median recurrence-free survival (range) = 12 months (1–99 months).

## Data Availability

The reader can access the raw data supporting the conclusions of the study by directly contacting the corresponding author.
